# Effect of Silica Xerogel Functionalization on Intensification of *Rindera graeca* Transgenic Roots Proliferation and Boosting Naphthoquinone Production

**DOI:** 10.3390/life14010159

**Published:** 2024-01-22

**Authors:** Kamil Wierzchowski, Bartosz Nowak, Mateusz Kawka, Katarzyna Sykłowska-Baranek, Maciej Pilarek

**Affiliations:** 1 Faculty of Chemical and Process Engineering, Warsaw University of Technology, Waryńskiego 1, 00-645 Warsaw, Poland; kamil.wierzchowski@pw.edu.pl (K.W.); bartosz.nowak@pw.edu.pl (B.N.); 2 Department of Pharmaceutical Biology, Faculty of Pharmacy, Medical University of Warsaw, Banacha 1, 02-097 Warsaw, Poland; mateusz.kawka@wum.edu.pl (M.K.); katarzyna.syklowska-baranek@wum.edu.pl (K.S.-B.)

**Keywords:** transgenic (hairy) roots, xerogel, in situ extraction, plant secondary metabolites

## Abstract

Secondary metabolites derived from plants are recognized as valuable products with several successful applications in the pharmaceutical, cosmetic, and food industries. The major limitation to the broader implementation of these compounds is their low manufacturing efficiency. Current efforts to overcome unprofitability depend mainly on biotechnological methods, especially through the application of plant in vitro cultures. This concept allows unprecedented bioengineering opportunities for culture system modifications with in situ product removal. The silica-based xerogels can be used as a novel, porous biomaterial characterized by a large surface area and high affinity to lipophilic secondary metabolites produced by plant tissue. This study aimed to investigate the influence of xerogel-based biomaterials functionalized with methyl, hydroxyl, carboxylic, and amine groups on *Rindera graeca* transgenic root growth and the production of naphthoquinone derivatives. The application of xerogel-based scaffolds functionalized with the methyl group resulted in more than 1.5 times higher biomass proliferation than for reference untreated culture. The naphthoquinone derivatives’ production was noted exclusively in culture systems supplemented with xerogel functionalized with methyl and hydroxyl groups. Applying chemically functionalized xerogels as in situ adsorbents allowed for the enhanced growth and productivity of in vitro cultured *R. graeca* transgenic roots, facilitating product isolation due to their selective and efficient accumulation.

## 1. Introduction

The plant kingdom is an inexhaustible natural and renewable source of organic compounds with a variety of proven benefits, e.g., biological activity. More than 80% of the world’s human population uses plants or plant-derived agents for health care, and almost half of the medical prescriptions that exist in the developed world originate from plants [1]. According to the World Health Organization, of the 252 basic and essential drugs, 11% are exclusively derived from plants [2]. Thus far, between 35,000 and 70,000 species of plants have been screened for their medicinal properties. Most biosynthesized compounds have applications or could potentially be used as bioactive ingredients in drugs or cosmetics [3,4,5,6,7,8]. The extensive bioactivity of secondary plant metabolites causes the high potential for their practical and prospective applications in the pharmaceutical, biotechnological, cosmetic, or food industries [9,10].

Most often, concentrations of many bioactive compounds in plant biomass harvested from natural resources are rather low, which makes obtaining the plant-derived agents economically unprofitable [11]. Furthermore, the efficient production of plant-derived therapeutics from natural sources is often restricted by seasonal variability and geographical, ecological, or political restrictions [12]. The application of modern biotechnological techniques, i.e., in vitro cultures of plant organs, like roots or shoots, allows the maintenance of plant biomass independently from the aspects mentioned above, but with plentiful options of parameters optimization and the scaling-up of bioprocess, it allows the exceeding of productivity observed in wild plants [13,14,15]. One of the practical bioengineering techniques widely recognized as a high-potential solution for the intensification of secondary plant metabolite production is in situ extraction [16,17,18,19]. Moreover, such a technique also provides a favorable microenvironment for the de novo production of secondary metabolites, which do not occur in the natural environment [20,21,22,23].

In the field cultivation of plants, as well as in plant biomass culture performed in laboratory conditions, the improvement of plant biomass growth and the increase of secondary metabolite production can be achieved by the addition of various chemical molecules like plant growth regulators, inductors, retardants, or elicitors [24,25,26,27,28]. These treatments can be supported by bioengineering enhancements of culture systems like in situ product removal, which was recognized for its high effectiveness among the leading techniques in plant biotechnology. Several experimental applications revealed that the supplementation of plant in vitro culture systems with additional phases (liquid or solid) capable of accumulating plant secondary metabolites results in a remarkable increase in their productivity.

As we demonstrated previously [14,23], the application of MTMS-based xerogel materials (with methyl groups on the surface) can significantly enhance plant secondary metabolism with preserved growth ability. Another key aspect of the interaction between the plant biomass and applied biomaterial is the chemical structure of the latter, including the presence of specific chemical residues or substituents [29,30,31,32]. The impact of individual organic groups in the structure of experimental xerogel materials on plant biomass growth and secondary metabolite production is still not fully known. Due to that, in this study, we individually and independently introduced several chemical groups to the surface of silica-based xerogel as biomaterial that is non-toxic against plant biomass used in our previous studies [14,23]. The TEOS-based xerogel was used as a basic material for the implementation of functional groups on the surface of silica xerogel. The TEOS-based xerogel allows for the easy attachment of precursors of other chemical groups due to the presence of reactive -OH groups on its surface [33,34,35].

This research aimed to recognize the effects of silica TEOS-based xerogel functionalization on transgenic root proliferation and secondary metabolite production. In this case, the influence of four silica xerogel-based biomaterials independently functionalized by various groups: (i) methyl (-CH_3_), (ii) hydroxyl (-OH), (iii) carboxylic (-COOH), and (iv) amine (-NH_2_), on the proliferation of *Rindera graeca* transgenic roots and production of naphthoquinones was quantitatively determined. Also, the effect of chemically functionalized xerogel on the morphology of in vitro-maintained transgenic roots was qualitatively analyzed.

## 2. Materials and Methods

### 2.1. TEOS Xerogel Synthesis

Tetraethyl orthosilicate (TEOS 98%, Merck, Poznań, Poland) was mixed in a polypropylene container with pure ethanol (Stanlab, Lublin, Poland). Aqueous solution of 0.01 M oxalic acid (Merck, Poznań, Poland) was added to start the hydrolysis. After 1 h of vigorous mixing, 1 M ammonium (Eurochem BGD, Tarnów, Poland) aqueous solution was added to shift the pH of the solution and initiate the polycondensation. The solution was mixed for 3 min and left at room temperature for gelation. Approximately 1 h after gelation, a small amount of ethanol was added on top of the gel to avoid a foil formation. The volumetric ratio of TEOS: ethanol: 0.01 M oxalic acid: 1 M ammonia equals to 1:2:0.6:0.6.

The obtained wet gel was transferred to larger containers and subjected to solvent exchange twice for pure ethanol and three times for n-hexane (Stanlab, Lublin, Poland). Finally, samples of gels were dried utilizing the ambient pressure method (APD) at 60 °C for approximately 4 days and next at 100 °C for 2 h to remove any residual solvent. Obtained materials were used for hairy root cultures as materials with hydroxy-groups (-OH).

### 2.2. TEOS-Based Xerogel Functionalization

TEOS wet gels, obtained as described above, were used as a basis for the preparation of further materials via surface functionalization. The total volume of modifying solutions equaled two times the volume of modified wet gel.

Before introducing amine (-NH_2_) groups via (3-aminopropyl)triethoxysilane (APTES 99%, Merck, Poznań, Poland), TEOS-based wet gels were exchanged into pure n-hexane. The modification mixture contained 5% of APTES in n-hexane, as a higher concentration or alcohol addition led to the formation of a sol–gel layer on the wet gel’s surface. After 24 h, the modified gels were rinsed three times with n-hexane.

APTES-modified wet gels were further used to obtain the carboxylic functional group (-COOH). Gels were subjected to solvent exchange with tetrahydrofuran (THF, butylene oxide anhydrous, Merck, Poznań, Poland) and then with succinic anhydride (SuAc, Merck, Poznań, Poland) solution (4.0 g SuAc/60 mL THF) for 24 h. After functionalization, modified gels were exchanged for n-hexane three times.

Methylation was performed via transferring ethanol-soaked TEOS gel into chlorotrimethylsilane (TMCS ≥ 98%, Merck, Poznań, Poland) solution for 24 h. The utilized volumetric ratio of TMCS:n-hexane:ethanol was equal to 15:55:30. After modification, the solution was exchanged three times with pure n-hexane.

All functionalized gels were dried using the same APD method procedure as described in Section 2.1.

### 2.3. Xerogel Characterization

All xerogels were measured and weighed to designate the apparent density and mass gained during functionalization, which was used to calculate the mass composition of materials. The volume shrinkage (*V*_s_) accompanying APD was calculated as the difference in gel volume before (wet) and after (dry) drying from the equation:

*V*_s_ = 100% × (*V*_wet_ − *V*_dry_) × *V*_dry_^−1^ (%) (1)

Basic materials characterization included SEM microscopy (TM1000, Hitachi, Tokyo, Japan) for structural analysis and FT-IR spectroscopy (ATR FT-IR Nicolet iS20, Thermo Fisher Scientific, Waltham, MA, USA) for chemical analysis. In the case of SEM imaging, samples of xerogels were sputtered with a conductive chromium and gold nanolayer.

The water-in-air contact angle was determined using a sessile drop method (OCA 25 goniometer, DataPhysics Instruments, Filderstadt, Germany). For each sample, 10 µL volume droplets on five different spots were placed at the designated contact angle. For each droplet, the contact angle measurement was repeated three times to eliminate the influence of oscillation.

Five samples of each xerogel, the previously measured weight of each piece, were put in olive or rapeseed oil for 24 h to determine the adsorption capacity (*AC*) of materials. The values of *AC* were estimated as a mass of adsorbed liquid per gram of xerogel according to the following equation:

*AC* = (*m*_wet_ − *m*_dry_) × *m*_dry_^−1^ (g_oil_/g_material_^−1^) (2)

where *m*_wet_ is the xerogel weight after the sorption of tested oil and *m*_dry_ is the xerogel weight before the sorption procedure.

### 2.4. Transgenic Roots Biomass

The *Rindera graeca* transgenic root line [36] has been applied. To maintain *R. graeca’s* hairy roots, 50 mL of DCR medium free of any plant growth regulator (PhytoTech Labs, Inc., Lenexa, KS, USA) in 250 mL Erlenmeyer flasks was poured. The transgenic roots were cultured at 24 °C in the dark on an oscillatory shaker ISS-7100 (Jeio Tech, Billerica, MA, USA) at 105 rpm. Subculturing of hairy roots was carried out every 28th day.

### 2.5. Cultures of Transgenic Roots on Xerogels

Cultures of *R. graeca* transgenic roots supported with micronized (fragments of approximately 1 mm diameter) functionalized xerogel containing various chemical groups, i.e., (i) methyl (-CH_3_), (ii) hydroxyl (-OH), (iii) carboxylic (-COOH), and (iv) amine (-NH_2_) substituents, were separately performed in 250 mL Erlenmeyer flasks filled by 50 mL of DCR medium free of plant growth regulators. Next, all culture systems were sterilized in an SMS80 autoclave (SMS, Góra Kalwaria, Poland) for 25 min at 121 °C and cooled to room temperature. For each culture system, 1 g of 28-day-old *R. graeca* hairy roots (as an inoculum) and 1 g of xerogel were added. As the reference system, the culture without any xerogel was performed. All culture systems were agitated on the oscillatory shaker at 105 rpm and 24 °C for 28 days in darkness. All cultures were carried out in seven replicates, i.e., seven Erlenmeyer flasks filled with transgenic roots and xerogel materials were used to conduct cultures in each culture system.

After 28 days, *R. graeca* roots were collected from Erlenmeyer flasks and their fresh weight was measured. Samples of transgenic roots, xerogels, and the culture medium (filtered using a 0.2 µm syringe filter) were separated and individually stored in a freezer at −20 °C. Next, all hairy roots and xerogels were lyophilized in ALPHA 1–4 LSC freeze dryer (Martin Christ Gefriertrocknungsanlagen GmbH, Osterode am Harz, Germany). N-hexane (Merck, Poznań, Poland) was applied for naphthoquinone extraction from the postculture medium and from lyophilized and micronized transgenic root biomass. The HPLC-grade methanol (Merck, Poznań, Poland) was used for naphthoquinones extraction from lyophilized xerogels. All samples were treated in a Sonorex ultrasonic scrubber (Bandelin, Berlin, Germany) to enhance the extraction efficiency until the solvent color faded. Obtained extracts were dried under reduced pressure (0.1 bar) using Rotavapor^®^ R-100 (Buchi, Flawil, Switzerland) and stored in a freezer at −20 °C. A detailed procedure used for secondary metabolite extraction from *R. graeca* transgenic roots is available in previous reports, e.g., in [14].

### 2.6. Phytochemical Analysis

Before analytical procedures, dried extracts from biomass, xerogels, and culture medium were mixed with methanol (HPLC-grade) (Merck, Poznań, Poland). Redissolved samples of extracts were chromatographically analyzed by a reversed-phase HPLC technique supported by the DIONEX 3000 HPLC system (Thermo Scientific, Waltham, MA, USA) equipped with an EC Nucleosil 120-7 ODS packed column (250 × 4.6 mm) filled by 7 μm particles with 120 Å pores (Macherey-Nagel, Allentown, PA, USA). A UVD 340S diode-array detector (Thermo Scientific, Waltham, MA, USA) was applied for qualitative and quantitative detection of chemical compounds. The chromatographic separation was performed using a gradient elution of acetonitrile (60–80%) and 0.04 M orthophosphoric acid (40–20%) at a flow rate of 1.5 mL min^−1^. Eluent absorbance was measured at four wavelengths: 215, 237, 350, and 436 nm. The concentrations of naphthoquinones, i.e., deoxyshikonin and rinderol, in extracts were quantitatively determined by the analysis of specified peaks at 215 nm (deoxyshikonin) and 237 nm (rinderol) wavelengths on chromatograms following the standard external method. Referenced deoxyshikonin and rinderol standards were used for qualitative identification of naphthoquinones peaks and calibration of the curve for quantitative analysis.

### 2.7. Mathematical Methods

The proliferation of transgenic roots during 28 days of culture was characterized by the fresh biomass (*FB*_28d_) increase, which has been estimated using the following equation:

*FB*_28d_ = *m*_28d_ × *m*_0d_^−1^ (−) (3)

where *m*_28d_ is the transgenic root fresh weight after 28 days of culture and *m*_0d_ is the transgenic root fresh weight inoculum.

The values of dry biomass (*DB*_28d_) increase were calculated according to the following equation:

*DB*_28d_ = *d*_28d_ × *d*_0d_^−1^ (−) (4)

where *d*_28d_ is the transgenic root dry weight after 28 days of culture and *d*_0d_ is the transgenic root dry weight inoculum.

The yield of naphthoquinones (i.e., deoxyshikonin and rinderol) production per dry biomass weight (*Y*_P/X_), i.e., 1.0 g of dry biomass, has been determined based on the following equation:

*Y*_P/X_ = *m*_p_ × (*d*_28d_ − *d*_0d_)^−1^ (g g_DW_^−1^) (5)

where *m*_p_ is the weight of naphthoquinones (i.e., deoxyshikonin and rinderol) after 28 days of culture.

For characterization of the growth rate of transgenic roots on xerogels, the values of specific growth rate (*μ*) have been calculated as follows:

*µ* = (ln *m*_28d_ − ln *m*_0d_) × (_Δ_*t*)^−1^ (h^−1^) (6)

where *t* is the time of culture.

### 2.8. Statistical Analysis

The statistical differences between results obtained for various culture systems concerning the xerogels characterization and the root biomass cultures were designated using one-way analysis of variance (ANOVA) with post hoc Tukey’s testing. For normal distribution and variance homogeneity, the Shapiro–Wilk normality test and Bartlett’s homoscedasticity test were applied, respectively, demonstrating that the ANOVA test conditions were fulfilled for all samples tested. The Student’s *t*-test was used for the rest of the statistical analysis. A *p*-value not higher than 0.01 was considered significant in all experiments.

## 3. Results

### 3.1. Characterization of Xerogels 

**Figure 1:**
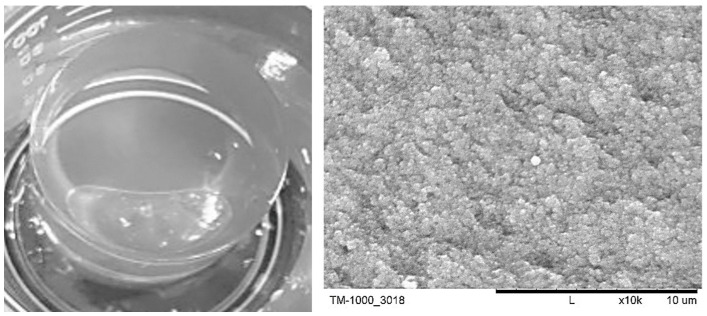
Morphology of TEOS-based wet gel (**left**) and SEM image of TEOS-based xerogel surface (**right**).

The TEOS-based materials used both directly for the cultivation of transgenic roots and for further functionalization were characterized by a uniform pore morphology of nanometric size (Figure 1), with a well-crosslinked and chemically uncomplicated structure of silicon oxide [37]. The small size of the pores results in the creation of high capillary pressure, which positively affects the kinetics of sorption, including the extraction of secondary metabolites [14].

**Figure 2:**
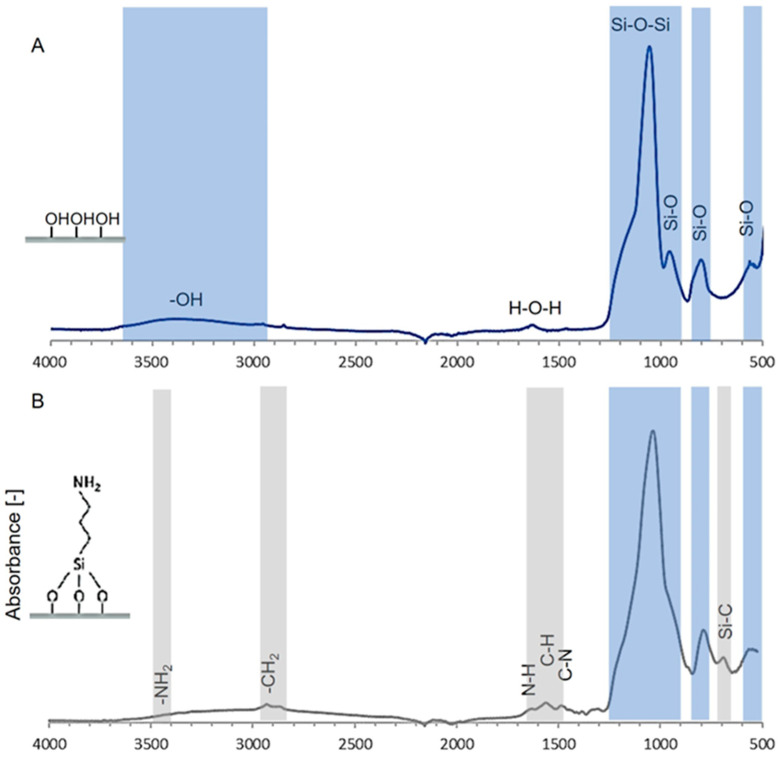

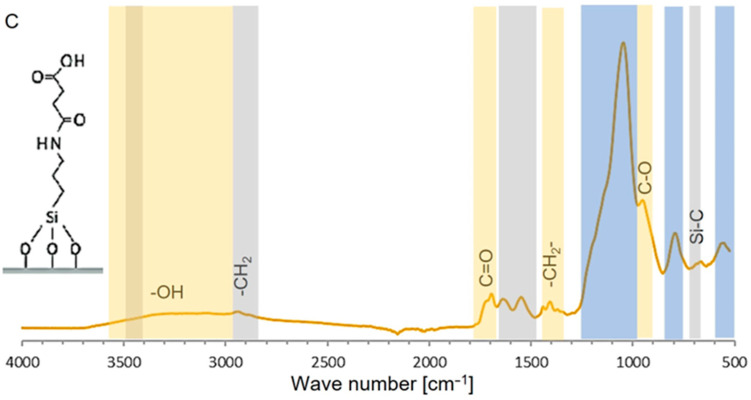
FT-IR spectra of studied materials (top to bottom): TEOS-based xerogel (**A**), after APTES functionalization (**B**), and after APTES and succinic anhydride functionalization (**C**).

**Figure 3:**
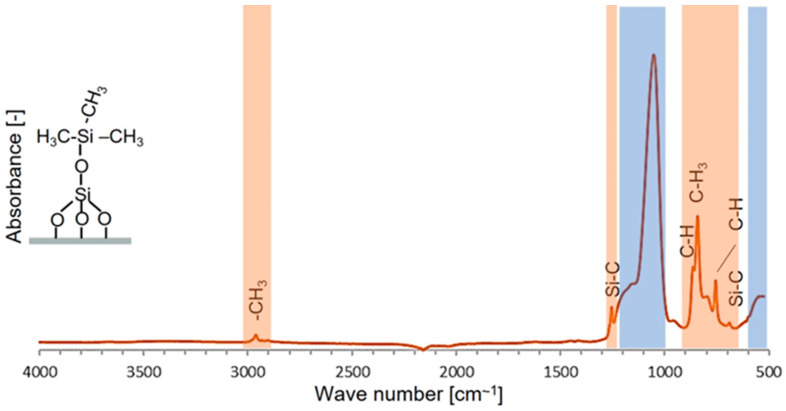
FT-IR spectrum of TMCS-functionalized TEOS-based xerogel.

FT-IR spectra of investigated xerogels are presented in Figure 2 and Figure 3. The curve in Figure 2A shows the typical silicon oxide structure of the TEOS-based material. Siloxane bonds building the xerogel skeleton are visible in peaks at 560 cm^−1^–stretching Si-O, 780 cm^−1^–bending Si-O, and 960 cm^−1^–stretching Si-O or Si-OH bonds. The double-peak at around 1080 and 1040 cm^−1^ describes the vibration of Si-O-Si asymmetric and symmetric, respectively. Reactive hydrogen bonding is visible as a broad peak at 3500–3000 cm^−1^ and is accompanied by a smaller peak at 1640 cm^−1^, corresponding to -OH stretching and bending modes, respectively [38,39].

TEOS modification with APTES (Figure 2B) is based on creating additional siloxane bonds between -OH groups in the gel skeleton and alkoxide groups of the siltation agent, thus lowering the intensity of 3500–3000 cm^−1^ and 960 cm^−1^ peaks. The propyl chain attached to the silicon atom is visible through the additional peak at 690 cm^−1^–Si-C bond, as well as CH_2_ groups in 2980 cm^−1^. The peak at 1565 cm^−1^ represents the C-N stretching bond, followed by the imine group at 1655 cm^−1^ and a less visible peak of -NH_2_ around 3500 cm^−1^ [40].

After the reaction of APTES with SuAc (Figure 2C), the intensity, peak width, and positions of vibrational modes around 1655 and 1565 cm^−1^ changed, and a new mode is visible around 1405 cm^−1^. These two vibrational modes are typical signatures of amide bonds formed by the succinylation of amino groups, as reported previously [41]. A peak at 1621 cm^−1^ is often assigned to the -CO-NH- bond [42]. While peaks at approximately 1723 cm^−1^–C=O and -OH broad peaks at 3500–3000 cm^−1^ arise from terminal carboxyl groups, the band at 1445 cm^−1^ is due to the methylene deformation mode of the propyl group [43].

The FT-IR spectrum of TEOS-based alcogel functionalization with tri-methyl groups is shown in Figure 3. In addition to the bonds typical of the siloxane network, xerogel has additional groups characteristic of the modifier. The Si-C bond is visible as new peaks at 1275 cm^−1^ and 690 cm^−1^ [44,45]. The presence of -CH_3_ groups is evidenced by peaks between 3000 and 2800 cm^−1^ (2939 cm^−1^–ν_as_ C-H methoxy group and 2842 cm^−1^–ν_s_ C-H methoxy group) and an extensive triple group of peaks in the range of 850–750 cm^−1^ [46,47].

**Figure 4:**
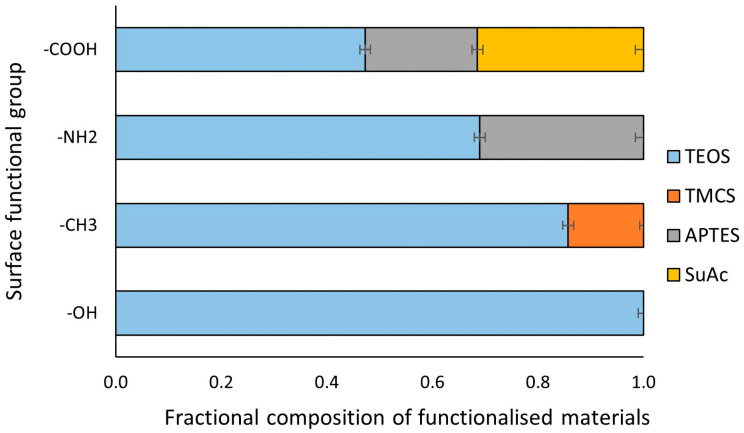
Mass fractional composition of studied functionalized silica-based xerogels.

Tracking changes in the mass of the framework (i.e., gel after drying) allows us to assess functionalization in terms of the composition of individual chemical structures. Figure 4 shows the fractional share of xerogels formed by combining chemical compounds. The share of TMCS in the structure of the methylated xerogel is approximately 14.3%, which, however, allows for the high hydrophobicity of the material, which will be presented later.

The combination of the TEOS-based network with APTES occurs with higher mass efficiency despite using a lower concentration in the modifying mixture, which is more than 30%. The subsequent modification of SuAc results in a material that consists of TEOS:APTES:SuAc in 47.3:21.3:31.4, respectively.

The results in Figure 4 and the FT-IR curves (i.e., Figure 2 and Figure 3) indicate precise functionalization and obtaining xerogels with the hydroxyl, methyl, amino, and carboxyl groups required for research.

**Figure 5:**
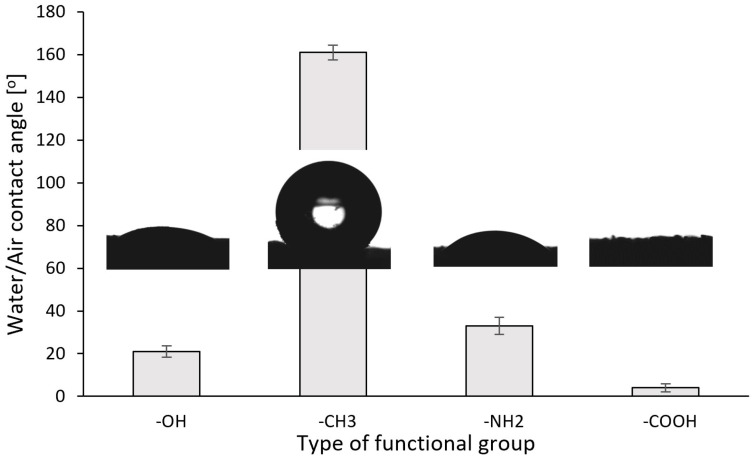
Water/air contact angle with corresponding sessile drop photos of studied xerogels.

Changing the functional group of the surface affects the value of the initial contact angle of water-in-air, as shown in Figure 5. Unfunctionalized xerogel from TEOS has a slightly hydrophilic character, giving a contact angle of approximately 21°. Despite being the smallest among the presented materials’ mass fraction of the modifier, the connection of tri-methyl silane results in strongly hydrophobic properties of TEOS/TMCS and a contact angle close to 160° [48]. Compared to TEOS, the APTES functionalized material has a slightly higher contact angle, reaching 33°. Although the -NH_2_ group is hydrophilic, it should be remembered that it is connected to the siloxane network through the propane chain [49]. The most hydrophilic samples, for which almost instantaneous absorption of drops was observed, are APTES/SuAc-functionalized xerogels with carboxyl surface groups.

**Figure 6:**
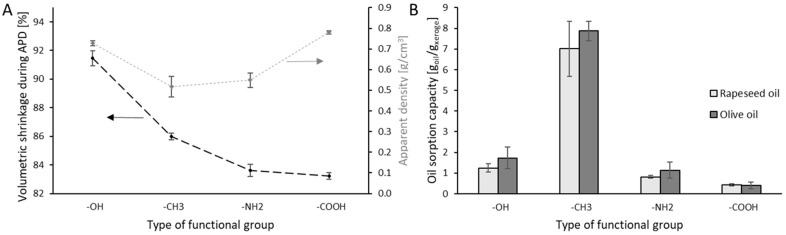
Volumetric shrinkage during APD and apparent density of xerogels with different functional groups (**A**). Oils’ sorption capacity of studied materials (**B**).

The use of xerogel as a sorbent for secondary metabolites depends on the porous nature of these materials. However, during the drying process under atmospheric pressure (APD), the porous nature is reduced due to capillary force. The porosity value helps understand the morphology’s effect on sorption capacity. However, it requires determining the skeletal density of the material, which changes with its chemical composition. TEOS-based xerogels will exhibit different skeletal densities after functionalization. Therefore, it was decided to present the results of apparent density and volume shrinkage (Figure 6A) as an alternative.

After drying, the obtained xerogels were tested for sorption capacity towards two vegetable oils (Figure 6B), i.e., olive and rapeseed. Direct sorption capacity tests on exemplary naphthoquinones were not possible due to their limited availability. In such cases, vegetable oils serve as a suitable substitute due to their chemical resemblance, including branched structure, presence of aromatic rings, and similar functional groups. It is important to note that the test liquids used in this study are a mixture of organic compounds [50]. Noteworthy is the higher sorption capacity of olive oil than rapeseed oil obtained for most samples. According to Liu et al. [51], this difference may be due to the density of the sorbed liquid, which is similar (approximately 0.91 g/cm^3^) in the case of tested oils. Although the exact reasons for this variation are not entirely clear, the higher viscosity or higher content of saturated and mono-unsaturated fatty acids in the olive oil composition [52] could be a possible explanation of the effect observed. Moreover, the porous structure can absorb various fractions of organic liquids with different efficiencies and at different rates, as demonstrated by sorption tests of crude oil [53]. Designated sorption capacity greatly exceeded the amount of produced secondary metabolites, which will be discussed in the next section.

The highest values of sorption capacity were obtained for methylated xerogel (Figure 6B). This is related to the oleophilicity of the functional groups and the low value of apparent density (Figure 6A). The TMCS groups present in the framework pores after functionalization has a minor (compared to the other tested modifications) steric hindrance, which does not cause clogging of the pore volume. Additionally, repulsive interactions between methyl groups prevent the pores from collapsing during APD, resulting in a lower volume shrinkage than for unmodified TEOS xerogel [54,55].

The APTES and APTES/SuAc modified xerogel show a slightly, approximately 2%, lower volumetric shrinkage than the TMCS-modified xerogel. Nonetheless, the absorption capacity of the oils is significantly lower. Despite its apparent density value being similar to TEOS/TMCS, xerogel with amine groups exhibits approximately eight times lower oil sorption capacity, indicating less oleophilic character of the material. The lowest values of sorption capacity recorded for xerogel with carboxyl groups may be related to the partial clogging of the pore volume of the TEOS framework by the chains of the modifying factors used. The high apparent density of the obtained material also indicates such an effect.

### 3.2. Effect of Xerogels on R. graeca Transgenic Root Cultures

#### 3.2.1. *R. graeca* Transgenic Root Morphology

**Figure 7:**
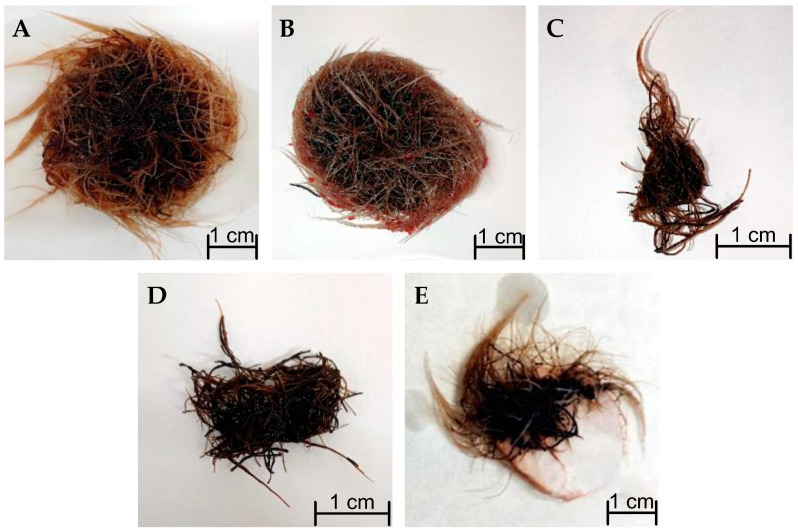
Morphology of *R. graeca* transgenic roots cultured in systems containing xerogels functionalized by various chemical groups: hydroxyl (-OH) (**A**), methyl (-CH_3_) (**B**), amine (-NH_2_) (**C**), carboxylic (-COOH) (**D**), and in the reference culture system without any xerogel (**E**).

The morphology of *R. graeca* transgenic roots harvested after 28 days from cultures supported with xerogel functionalized by various chemical groups is presented in Figure 7. Hairy roots in culture systems containing xerogels with methyl and hydroxyl groups were highly branched and strongly condensed (Figure 7A,B). Furthermore, a substantial amount of young transgenic roots, which are seen as bright roots on the edges of the hairy root bunch, was observed for these culture systems. The young transgenic roots were also easy to see for the reference culture system, but their amount was significantly lower than for systems containing xerogels with methyl and hydroxyl groups (Figure 7E). The lack of young transgenic roots and darkened biomass was observed for culture systems containing xerogels supported with carboxylic and amine groups (Figure 7C,D).

#### 3.2.2. Fresh Biomass Relative Growth (*FB*_28d_)

**Figure 8:**
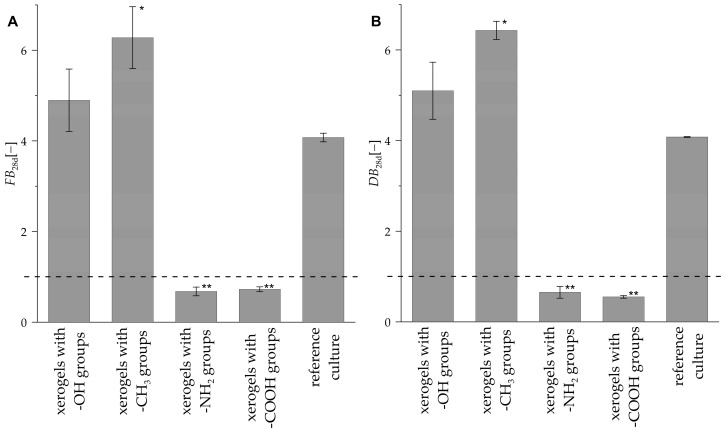
Values of *FB*_28d_ (**A**) and *DB*_28d_ (**B**) characterizing *R. graeca* transgenic root proliferation in systems containing xerogels functionalized by various chemical groups and in reference culture systems. The asterisks indicate the treatments with values statistically different from the reference culture, with *p* < 0.01 (*) and *p* < 0.001 (**) determined by the ANOVA test.

The *FB*_28d_ values characterizing the *R. graeca* transgenic root proliferation in systems containing xerogels functionalized by methyl, hydroxyl, carboxylic, amine groups, and in the reference culture system without any xerogel are presented in Figure 8A. The highest value of *FB*_28d_ (i.e., 6.28) was noticed for the culture system containing xerogel functionalized by methyl groups, and it was more than 54% higher than the *FB*_28d_ value determined for the control culture system (i.e., 4.07). A higher value of *FB*_28d_ than for the reference culture system was observed for the culture system containing xerogels functionalized by hydroxyl groups (i.e., 4.89). In the case of the culture system containing xerogels functionalized by carboxylic and amine groups, the *FB*_28d_ values were less than 1, which indicates a lack of transgenic root proliferation in the culture system containing xerogels functionalized by this kind of substituents.

#### 3.2.3. Dry Biomass Relative Growth (*DB*_28d_)

The *DB*_28d_ values, characterizing the fold change of dry weight of the *R. graeca* transgenic roots in systems containing xerogels functionalized by methyl, hydroxyl, carboxylic, amine groups, and the reference culture without xerogel, are all presented in Figure 8B. The highest value of *DB*_28d_ (i.e., 6.43) was observed for the culture system containing xerogel functionalized by methyl groups, and this value was more than 57% higher than the *DB*_28d_ value determined for the reference culture system (i.e., 4.08). A higher value of *DB*_28d_ than for the reference culture system was noticed for the culture system containing xerogels functionalized by hydroxyl groups (i.e., 5.10). For culture systems containing xerogels functionalized by carboxylic and amine groups, a decrease in transgenic root dry weight was observed, with the *DB*_28d_ values less than 1.

#### 3.2.4. Specific Growth Rate (*µ*)

**Figure 9:**
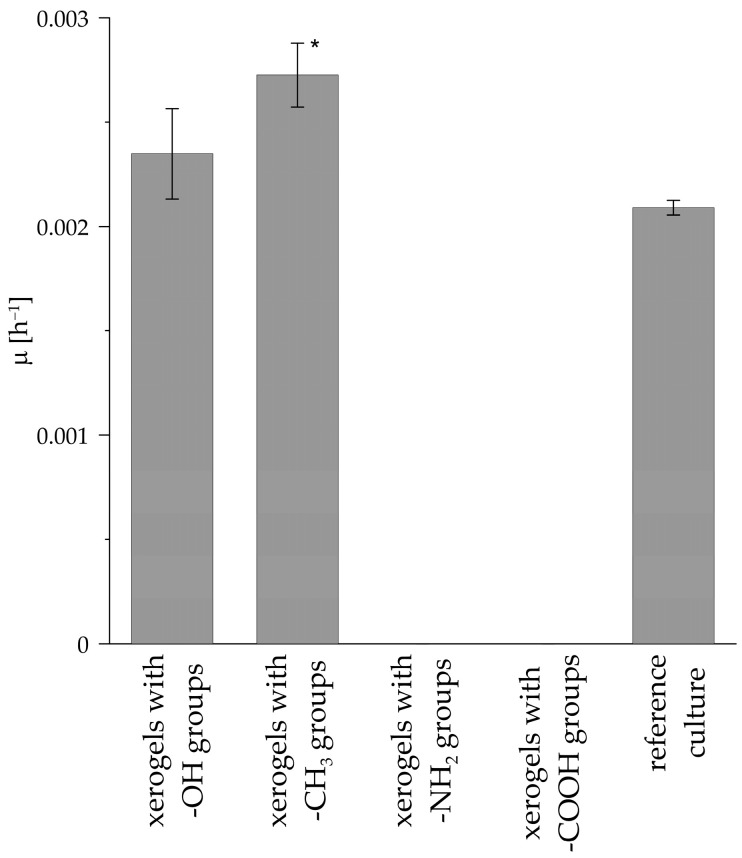
Values of *µ* characterizing the growth rate of *R. graeca* transgenic roots cultured in systems containing xerogels functionalized by various chemical groups and in reference culture systems. The asterisk indicates the treatments with values statistically different from the reference culture, with *p* < 0.001 (*) determined by the ANOVA test.

The *µ* values characterizing the growth rate of the *R. graeca* transgenic roots in systems containing xerogels functionalized by methyl, hydroxyl, carboxylic, amine groups, and in the reference culture without xerogel are presented in Figure 9. The highest value of *µ* (i.e., 0.0027 h^−1^) was noticed for the culture system containing xerogel functionalized by methyl groups, and this value was more than 30% higher than the *µ* value determined for the reference culture system (i.e., 0.0021 h^−1^). A higher value of *µ* than for the reference culture system was observed for the culture system containing xerogels functionalized by hydroxyl groups (i.e., 0.0023 h^−1^). For culture systems containing xerogels functionalized by carboxylic and amine groups, the *µ* values were less than 0, i.e., transgenic root growth was not observed for these culture systems. Values less than 0 were noted due to the decay of transgenic roots during the culture. This suggests that the transgenic roots were disintegrated in the very first days of culture.

#### 3.2.5. Naphthoquinones Production (*m*_p_)

**Figure 10:**
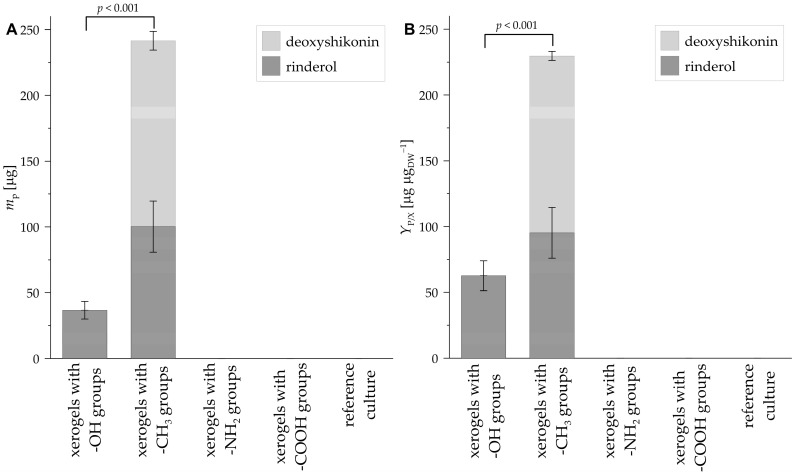
Mass of deoxyshikonin and rinderol produced by *R. graeca* transgenic roots (**A**) and values of *Y*_P/X_ characterizing the efficiency of deoxyshikonin and rinderol production (**B**) in culture systems containing xerogels functionalized by various chemical groups and in the reference culture. The *p*-value over the bracket was determined by the ANOVA test.

The *m*_p_ values, characterizing the mass of naphthoquinones (i.e., deoxyshikonin and rinderol) produced by the *R. graeca* transgenic roots in systems containing xerogels functionalized by methyl, hydroxyl, carboxylic, amine groups, and in the reference culture without xerogel, are presented in Figure 10A. The highest value of *m*_p_ was noticed for the culture system supported with xerogel functionalized by methyl groups, in which 141 µg of deoxyshikonin and 100 µg of rinderol were detected (i.e., 241 µg of naphthoquinones in total). For the culture system containing xerogel functionalized by hydroxyl groups, 37 µg of rinderol was detected. In the case of systems containing xerogels functionalized by carboxylic and amine groups, and in reference culture without xerogel, the amount of both naphthoquinones, i.e., deoxyshikonin and rinderol, did not reach the detection shoulder. In the studied systems, the entire mass of deoxyshikonin and rinderol produced by *R. graeca* transgenic roots was accumulated in xerogels. A lack of deoxyshikonin and rinderol was observed in samples from cultured medium or transgenic roots.

#### 3.2.6. Yield of Naphthoquinones Production per Unit Mass (*Y*_P/X_)

The *Y*_P/X_ values, characterizing the production efficiency of naphthoquinones (i.e., deoxyshikonin and rinderol) biosynthesized by the *R. graeca* transgenic roots in systems containing xerogels functionalized by methyl, hydroxyl, carboxylic, amine groups, and in the reference culture without any xerogel, are presented in Figure 10B. The highest value of *Y*_P/X_ was noticed for the culture system containing xerogel functionalized by methyl groups. For such a culture system, the *Y*_P/X_ values equaled 134 µg µg_DW_^−1^ for the deoxyshikonin production and 95 µg µg_DW_^−1^ for the rinderol production (i.e., 229 µg µg_DW_^−1^ in total) after 28 days of culture. For the culture system supported with xerogel functionalized by hydroxyl groups, the *Y*_P/X_ value was only determined for the rinderol production, and it equaled 63 µg µg_DW_^−1^ after 28 days of culture. In the case of systems with xerogels functionalized by carboxylic, amine groups, and in the reference culture without xerogel, the values of *Y*_P/X_ for both naphthoquinones, i.e., deoxyshikonin and rinderol production, equaled 0 µg, due to the lack of naphthoquinones in these systems.

## 4. Discussion

Four independently functionalized silica-based xerogels were characterized and tested as biomaterials for hairy root culture. Differences between the tested materials that may affect the obtained results of culture and the production of secondary metabolites should be addressed and discussed.

Among all tested xerogels, three exhibit hydrophilic characteristics, followed by limited organic liquid sorption capacity, and the methyl-functionalized one is superhydrophobic and lipophilic with a high sorption capacity towards organic liquid. The values of the water/air contact angle describing the xerogel wettability do not directly impact biomass growth and secondary metabolite production, as the highest values were observed for xerogel with -OH (hydrophilic) and -CH_3_ (hydrophobic) functional surface groups. The other two hydrophilic materials, containing amine and carboxylic groups, gave unsatisfactory results, as the lack of biomass growth and secondary metabolite production was noted.

It is crucial to consider the indirect effect of the wettability of materials. Hydrophilic xerogels can be filled with metabolic products but also with a water-based culture medium, making the pore fraction unavailable to naphthoquinones. Additionally, water sorption reduces capillary forces, acting as a suction pump for organic liquids and thus limiting sorption kinetics. This can significantly slow down the removal of metabolites from the surface of transgenic roots, ultimately leading to faster poisoning of the biomass. The TEOS/TMCS xerogel’s superhydrophobic nature (as shown in Figure 5) allows all pores to remain accessible, preventing the aforementioned effects from occurring.

**Figure 11:**
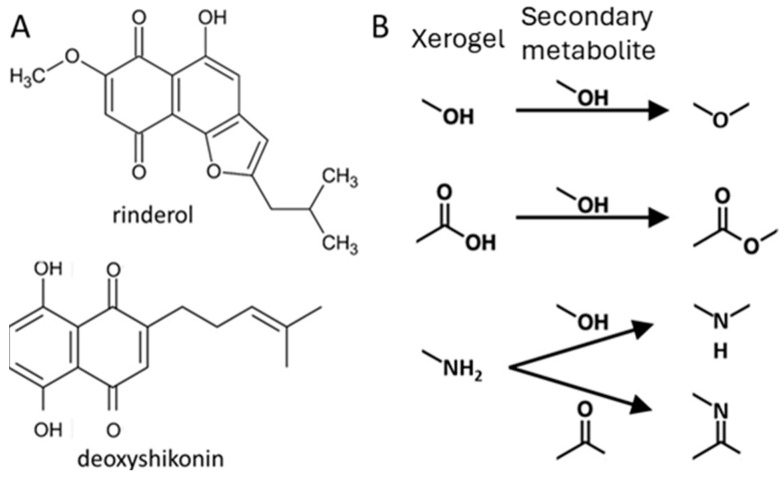
Rinderol and deoxyshikonin chemical structure (**A**). Possible chemical reactions between xerogel functional groups and secondary metabolites (**B**).

The test results indicate a positive effect of xerogel with methyl groups, which excludes chemisorption due to their low reactivity. However, it is important to note that the remaining functional groups are electron donors/acceptors, which could result in the formation of hydrogen bonds and irreversible chemical bonds with secondary metabolites (Figure 11). The literature data indicate the usefulness of amine-functionalized material for the sorption of organic compounds by chemical bonding rather than physisorption [49]. Chemical bonding occurs due to the presence of carboxyl and carbonyl groups in the designated naphthoquinones, as well as the methoxy group in the structure of rinderol. It is possible that chemisorption could prevent the recovery of produced metabolites from the pores of the material in the liquid-xerogel extraction process, which could explain the absence (or lower amount in the case of -OH) of products in these cultures. However, this theory should be dismissed due to the lack of a characteristic intense red color in the xerogel. Instead, it should be assumed that the materials used with amine and carboxyl groups inhibited root growth and production.

The occurrence of reactive groups on the surface of xerogels containing hydroxyl, carboxylic, and amine groups may have initiated a chemical reaction with the components of the culture medium, especially during sterilization at high temperatures and under increased pressure. Such hypothetical interactions between the culture medium and the tested materials could have affected the chemical structure of the xerogel, changed the composition of the culture medium, or even changed the pH level of the culture medium. This phenomenon seems likely due to the presence of organic compounds in the culture medium, i.e., vitamins and myoinositol, that could react with the functional groups of the xerogels. The interactions between culture medium compounds and functional groups of xerogels will be examined in further studies.

Another difference between functionalized materials is their surface charge. The dissociation of hydroxyl and carboxyl groups in water releases H+ ions, leading to a negative surface charge. Similarly, contact with water causes basic amine groups to protonate, resulting in a positive surface charge. Although the effect of surface charge on culture is not evident, it might alter it indirectly via changing the medium pH.

**Figure 12:**
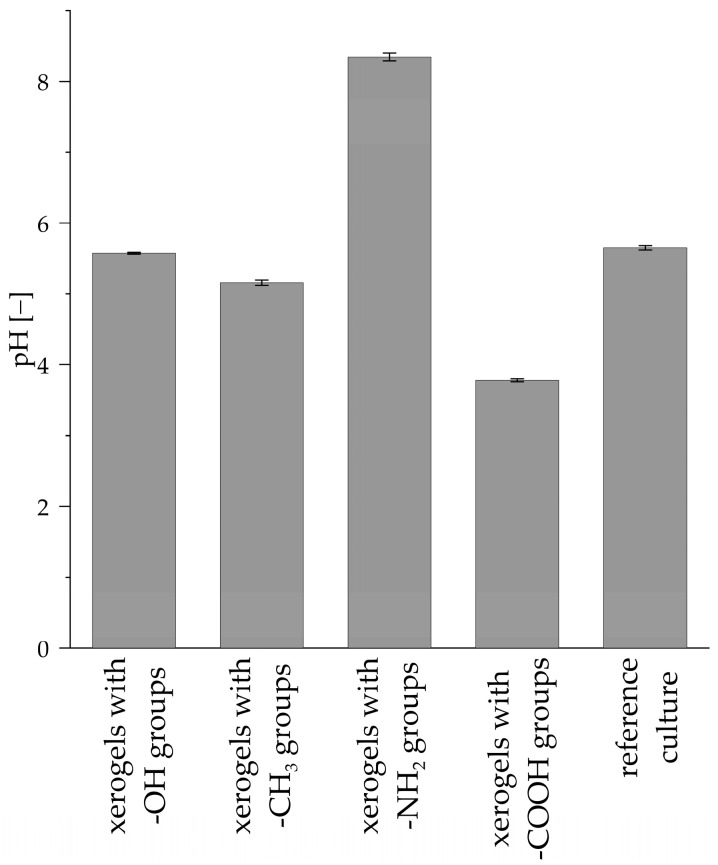
Values of pH level of the culture medium after 28 days culture of *R. graeca* transgenic roots.

Culture medium pH level measurements performed on the 28th day of the culture of *R. graeca* transgenic roots showed differences for APTES- and APTES/SuAc-modified TEOS-based xerogels (Figure 12). The pH level of the medium from culture systems containing xerogels functionalized with hydroxy and methyl groups was similar to the pH level characterizing the reference culture not supported with any xerogel. However, xerogels containing amide and carboxyl groups caused significant changes in pH levels, resulting in increased and decreased pH values, respectively. The presented phenomenon agrees with the functional group’s character, as the -NH_2_ exhibits a negative charge and alkaline character [56], while -COOH is characteristic for organic acids [57]. We hypothesize that the lack of transgenic root proliferation might be caused by the changes described above in the pH level of the culture medium. A negative surface charge effect is less probable as TEOS-based culture, with the presence of negative -OH group, gave far better results.

The effect of limited and only slightly shifting the pH level of the culture medium by methylated xerogel indicates that such biomaterial may be the most promising one for supporting *R. graeca* hairy root cultures. For methylated xerogel, both proliferation and secondary metabolite production was the most intensive among all tested xerogels, and also the production of deoxyshikonin, a pharmaceutically valuable bioproduct [58,59,60], was observed. The xerogel surface containing non-polar -CH_3_ groups resulted in a high organic liquid sorption capacity and high hydrophobicity [61]. High sorption capacity, caused by high capillary pressure in a well-developed mesoporous structure, may lead to fast sorption kinetics of secreted naphthoquinones. Such an effect may decrease the biological stress caused by the occurrence of extracellularly secreted toxic bioproducts in the neighborhood of the proliferating *R. graeca* transgenic roots. Furthermore, super hydrophobicity limits aqueous culture medium sorption and leaves an open-porous structure available for plant metabolites.

## 5. Conclusions

The present study demonstrated the effects of silica xerogel functionalization on the growth and secondary metabolite production in cultures of *R. graeca* transgenic roots. Surface modifications of TEOS-based xerogels with various organic functional groups (i.e., hydroxyl, methyl, amine, and carboxyl substituents) resulted in a specified metabolic response from cultured biomass of *R. graeca* hairy roots. The application of xerogels modified with hydroxyl and methyl substituents in cultures of *R. graeca* transgenic roots resulted in boosted naphthoquinones, i.e., deoxyshikonin and rinderol production, which was not observed in the reference culture not supplemented with any xerogel. The yield of naphthoquinone derivatives’ production values equaled 229 µg g_DW_^−1^ and 63 µg g_DW_^−1^ for culture systems containing xerogel functionalized by the methyl and the hydroxyl groups, respectively. In addition, the values of biomass increase in cultures containing xerogels modified with hydroxyl and methyl groups were higher than in the reference culture. More than 6-fold fresh biomass increase was noticed for culture systems containing xerogel functionalized with the methyl group. Contrary to these results, supplementation of culture systems with xerogel modified with amine and carboxyl groups limited (or even stopped) root growth and the production of secondary metabolites. Changes in the pH level of the culture medium caused by the properties of xerogel functionalized by amine and carboxyl groups and possible interactions between these xerogels with the culture medium were identified as the main reasons resulting in the lack of growth, disrupting the metabolic pathway for naphthoquinones biosynthesis, and even producing degeneration of in vitro cultured *R. graeca* transgenic roots.

To sum up, silica-based xerogels functionalized with hydroxyl and methyl substituents may be successfully applied for specified and directed intensification of transgenic root proliferation and for boosting naphthoquinone production in *R. graeca* hairy roots in vitro cultures.

## Data Availability

The data presented in this study are available on request from the corresponding author.
